# Supplementary material for: Expanding the repertoire of human tandem repeat RNA-binding proteins
Source: PLoS One. 2023 Sep 20;18(9):e0290890. doi: 10.1371/journal.pone.0290890 (PMC10511089; doi:10.1371/journal.pone.0290890)
Supplement: S1 File — (ZIP) [file pone.0290890.s001.zip › Supplementary Information/Table-S3.pdf]

Table S3: Full list of the Pfam domains located in trRBDpeps among the subsets studied in this work.

**Sequence-based subset (24)**		
**trRBDpep matches**	**Pfam code**	**Description**
6	PF00098	Zinc knuckle
3	PF00418	Tau and MAP protein, tubulin-binding repeat
2	PF01535	PPR repeat
1	PF15266	Domain of unknown function (DUF4594)
1	PF13865	C-terminal duplication domain of Friend of PRMT1
1	PF01280	Ribosomal protein L19e
1	PF00428	60s Acidic ribosomal protein
1	PF00076	RNA recognition motif. (a.k.a. RRM, RBD, or RNP domain)

**Sequence+Structure-based subset (128)**		
**trRBDpap matches**	**Pfam code**	**Description**
76	PF00076	RNA recognition motif. (a.k.a. RRM RBD or RNP domain)
27	PF00013	KH domain
5	PF00270	DEAD/DEAH box helicase
3	PF00565	Staphylococcal nuclease homologue
3	PF00271	Helicase conserved C-terminal domain
2	PF13516	Leucine Rich repeat
2	PF09011	HMG-box domain
2	PF08080	RNPHF zinc finger

2	PF02218	Repeat in HS1/Cortactin
2	PF01424	R3H domain
2	PF01271	Granin (chromogranin or secretogranin)
2	PF00880	Nebulin repeat
2	PF00505	HMG (high mobility group) box
2	PF00458	WHEP-TRS domain
2	PF00400	WD domain G-beta repeat
2	PF00307	Calponin homology (CH) domain
2	PF00041	Fibronectin type III domain
1	PF18360	Heterogeneous nuclear ribonucleoprotein Q acidic domain
1	PF15519	Linker between RRM2 and RRM3 domains in RBM39 protein
1	PF13893	RNA recognition motif. (a.k.a. RRM RBD or RNP domain)
1	PF13855	Leucine rich repeat
1	PF12287	Cytoplasmic activation/proliferation-associated protein-1 C term
1	PF12265	Histone-binding protein RBBP4 or subunit C of CAF1 complex
1	PF11835	RRM-like domain
1	PF11532	HnRNP M nuclear localisation signal
1	PF09371	Tex-like protein N-terminal domain
1	PF08777	RNA binding motif
1	PF08368	FAST kinase-like protein subdomain 2
1	PF08156	NOP5NT (NUC127) domain
1	PF08152	GUCT (NUC152) domain
1	PF08075	NOPS (NUC059) domain
1	PF07992	Pyridine nucleotide-disulphide oxidoreductase

1	PF07686	Immunoglobulin V-set domain
1	PF07528	DZF domain
1	PF05843	Suppressor of forked protein (Suf)
1	PF05835	Synaphin protein
1	PF04845	PurA ssDNA and RNA-binding protein
1	PF03096	Ndr family
1	PF02187	Growth-Arrest-Specific Protein 2 Domain
1	PF02029	Caldesmon
1	PF01602	Adaptin N terminal region
1	PF01423	LSM domain
1	PF00769	Ezrin/radixin/moesin family C terminal
1	PF00658	Poly-adenylate binding protein unique domain
1	PF00642	Zinc finger C-x8-C-x5-C-x3-H type (and similar)
1	PF00595	PDZ domain
1	PF00575	S1 RNA binding domain
1	PF00567	Tudor domain
1	PF00412	LIM domain
1	PF00396	Granulin
1	PF00249	Myb-like DNA-binding domain
1	PF00153	Mitochondrial carrier protein
1	PF00118	TCP-1/cpn60 chaperonin family
1	PF00085	Thioredoxin
1	PF00012	Hsp70 protein

Structure-based subset (67)		
**trRBDpep matches**	**Pfam code**	**Description**
7	PF00076	RNA recognition motif. (a.k.a. RRM, RBD, or RNP domain)
4	PF01248	Ribosomal protein L7Ae/L30e/S12e/Gadd45 family
3	PF00244	14-3-3 protein
3	PF00085	Thioredoxin
2	PF00056	lactate/malate dehydrogenase, NAD binding domain
2	PF00012	Hsp70 protein
1	PF20145	ARMET, N-terminal
1	PF18031	Ubiquitin carboxyl-terminal hydrolases
1	PF17835	NOG1 N-terminal helical domain
1	PF17136	Ribosomal proteins 50S L24/mitochondrial 39S L24
1	PF13691	tRNase Z endonuclease
1	PF10208	ARMET, C-terminal
1	PF09439	Signal recognition particle receptor beta subunit
1	PF08080	RNPHF zinc finger
1	PF08069	Ribosomal S13/S15 N-terminal domain
1	PF07749	Endoplasmic reticulum protein ERp29, C-terminal domain
1	PF06244	Coiled-coil domain-containing protein 124 /Oxs1
1	PF05300	MICOS complex subunit MIC19/MIC25
1	PF04597	Ribophorin I
1	PF03952	Enolase, N-terminal domain
1	PF03764	Elongation factor G, domain IV

1	PF03378	CAS/CSE protein, C-terminus
1	PF03357	Snf7
1	PF03308	Methylmalonyl Co-A mutase-associated GTPase MeaB
1	PF03144	Elongation factor Tu domain 2
1	PF02874	ATP synthase alpha/beta family, beta-barrel domain
1	PF02866	Lactate/malate dehydrogenase, alpha/beta C-terminal domain
1	PF02826	D-isomer specific 2-hydroxyacid dehydrogenase, NAD binding domain
1	PF02825	WWE domain
1	PF02803	Thiolase, C-terminal domain
1	PF02779	Transketolase, pyrimidine binding domain
1	PF02737	3-hydroxyacyl-CoA dehydrogenase, NAD binding domain
1	PF02629	CoA binding domain
1	PF02525	Flavodoxin-like fold
1	PF01775	Ribosomal proteins 50S-L18Ae/60S-L20/60S-L18A
1	PF01709	Transcriptional regulator
1	PF01509	TruB family pseudouridylate synthase (N terminal domain)
1	PF01423	LSM domain
1	PF01158	Ribosomal protein L36e
1	PF01090	Ribosomal protein S19e
1	PF00887	Acyl CoA binding protein
1	PF00864	ATP P2X receptor
1	PF00595	PDZ domain
1	PF00312	Ribosomal protein S15

1	PF00274	Fructose-bisphosphate aldolase class-I
1	PF00231	ATP synthase
1	PF00226	DnaJ domain
1	PF00180	Isocitrate/isopropylmalate dehydrogenase
1	PF00177	Ribosomal protein S7p/S5e
1	PF00011	Hsp20/alpha crystallin family
1	PF00006	ATP synthase alpha/beta family, nucleotide-binding domain
1	PF00005	ABC transporter
