# Supplementary material for: Expanding the repertoire of human tandem repeat RNA-binding proteins
Source: PLoS One. 2023 Sep 20;18(9):e0290890. doi: 10.1371/journal.pone.0290890 (PMC10511089; doi:10.1371/journal.pone.0290890)
Supplement: S1 File — (ZIP) [file pone.0290890.s001.zip › Supplementary Information/Table-S2.pdf]

Table S2: List of the 136 proteins experimentally determined as RNA binding proteins that are not predicted as repetitive or not constitute a main isoform.

A8MUS3	J3KPS0	P15924	P46776	P62316	Q04837	Q8N5K1	Q9NWU5
B0YIW6	J3KPS3	P16401	P46777	P62328	Q06830	Q8WUH6	Q9NZ45
B4DG01	J3KTL2	P18621	P46778	P62805	Q08J23	Q8WW12	Q9P0M9
B4DGP8	O43768	P23284	P46779	P62829	Q09666	Q92665	Q9UHR5
B4DNJ6	O60306	P23396	P46782	P62854	Q12955	Q96RR5	Q9UKM9
B4DSS8	O60812	P23528	P49792	P62857	Q15056	Q96T58	Q9UNP9
B4DT31	O60869	P24043	P54277	P62906	Q4U2R6	Q9BQ48	Q9UNZ2
B4DTG2	O75380	P24752	P60002	P62910	Q5H945	Q9BYD1	Q9UQ35
B4DVB8	O75821	P25789	P60468	P62942	Q5JTJ3	Q9BYD3	Q9Y221
B7Z4C8	P02511	P26373	P60866	P63173	Q5RKV6	Q9BYG3	Q9Y2S6
D3DQV9	P04040	P26885	P61081	P68036	Q5T1Z8	Q9GZT3	Q9Y2V2
D6W592	P04054	P30050	P61247	P80723	Q5VWC4	Q9GZZ1	Q9Y3D5
D6W5Y5	P06730	P30086	P61313	P82912	Q6IPX4	Q9H825	Q9Y3D9
G3XAD8	P07195	P31153	P61604	Q00266	Q6NW29	Q9H9G7	Q9Y3I0
G5E9Q2	P10599	P35555	P62241	Q00688	Q6P1L8	Q9NSD9	Q9Y520
G5EA30	P11171	P39023	P62263	Q02878	Q6ZN17	Q9NVS2	Q9Y606
H0YNJ6	P15531	P42677	P62266	Q03001	Q86WV7	Q9NW64	
