# Supplementary material for: Expanding the repertoire of human tandem repeat RNA-binding proteins
Source: PLoS One. 2023 Sep 20;18(9):e0290890. doi: 10.1371/journal.pone.0290890 (PMC10511089; doi:10.1371/journal.pone.0290890)
Supplement: S1 File — (ZIP) [file pone.0290890.s001.zip › Supplementary Information/Table-S1.pdf]

Table S1: List of the 219 RNA-binding tandem repeat proteins.

**Sequence repeat proteins (24)**							
P05387	P16989	P42704	P62633	Q01130	Q7L2E3	Q8NC51	Q9H7B2
P06748	P27816	P46783	P62995	Q01844	Q86V81	Q8ND56	Q9NUD5
P10636	P35637	P55265	P84098	Q13595	Q8IXT5	Q92804	Q9Y3X0
**Sequence + Structure repeat proteins (128)**							
A0AV96	P09622	P26368	P61978	Q13151	Q15269	Q8N5C6	Q96T37
O00425	P09651	P26583	P62314	Q13242	Q15291	Q8N6U8	Q99442
O00567	P09661	P26599	P62318	Q13243	Q15365	Q8NCA5	Q99729
O14828	P10645	P28799	P63244	Q13247	Q15366	Q8NDT2	Q9BQ67
O14979	P10809	P31483	P67809	Q13310	Q15427	Q8TDD1	Q9BY67
O15143	P11142	P31942	Q00341	Q13347	Q15434	Q8WXF1	Q9H095
O43390	P11940	P31943	Q00577	Q13409	Q16181	Q92597	Q9H6T0
O43903	P13489	P43243	Q01085	Q13884	Q53EP0	Q92688	Q9NR30
O60506	P14866	P47974	Q05682	Q14103	Q5BKZ1	Q92841	Q9NX07
O75494	P17844	P51114	Q08211	Q14240	Q6PKG0	Q92945	Q9NY93
O76094	P19338	P51911	Q08752	Q14247	Q6PUV4	Q96DH6	Q9NYY8
O94979	P20774	P51991	Q12849	Q14444	Q6ZT89	Q96EP5	Q9NZI8
P05455	P21291	P52272	Q12905	Q14498	Q7KZF4	Q96GQ7	Q9Y2K5
P07585	P22626	P52756	Q12926	Q14690	Q86U38	Q96I24	Q9Y4C8
P07814	P23246	P55795	Q13087	Q14847	Q8IUH3	Q96KC8	Q9Y678
P09429	P26038	P60842	Q13148	Q15032	Q8IWR0	Q96PK6	Q9Y6M1
**Structure repeat proteins (67)**							
O43175	P13639	P31946	P48735	P62277	Q16836	Q9BZE4	Q9Y2R9

O95900	P15170	P36542	P49411	P62308	Q7L0Y3	Q9H0J9	Q9Y3U8
P04792	P15559	P38646	P49591	P62424	Q8IVH4	Q9H444	Q9Y5K5
P04843	P25398	P39019	P52597	P62888	Q8NEJ9	Q9NQ94	Q9Y5M8
P06733	P25705	P40925	P53597	P63104	Q96A35	Q9NVA2	
P07108	P29401	P40926	P55060	Q01995	Q96CT7	Q9NX24	
P07237	P30040	P42765	P55145	Q02543	Q99572	Q9NX63	
P09972	P30101	P43897	P61221	Q13425	Q9BQ52	Q9P2W9	
P11021	P31689	P46781	P61981	Q13823	Q9BSH4	Q9UHX1	

All the proteins here listed are predicted as repetitive by at least one method, and their RNA binding sites were determined experimentally.
