# Supplementary figures and images for: Expanding the repertoire of human tandem repeat RNA-binding proteins
Source: PLoS One. 2023 Sep 20;18(9):e0290890. doi: 10.1371/journal.pone.0290890 (PMC10511089; doi:10.1371/journal.pone.0290890)

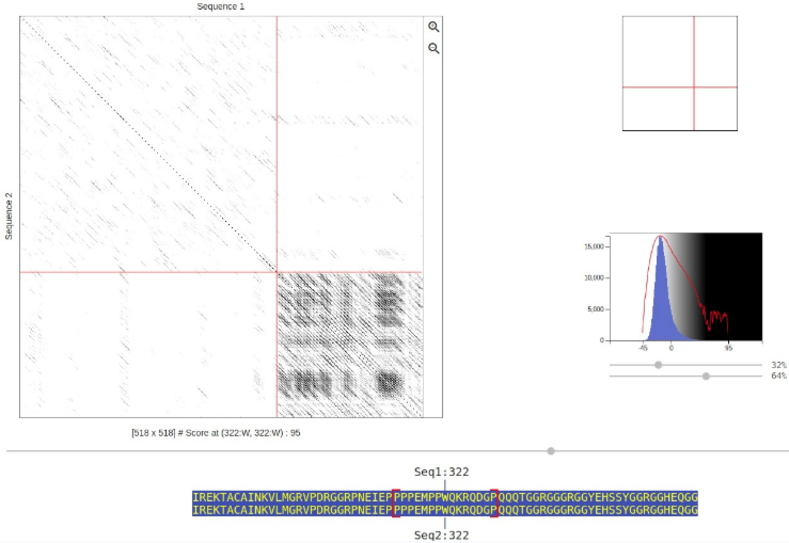

Supplement: S1 File — (ZIP) [file pone.0290890.s001.zip › Supplementary Information/Fig S2.tif]

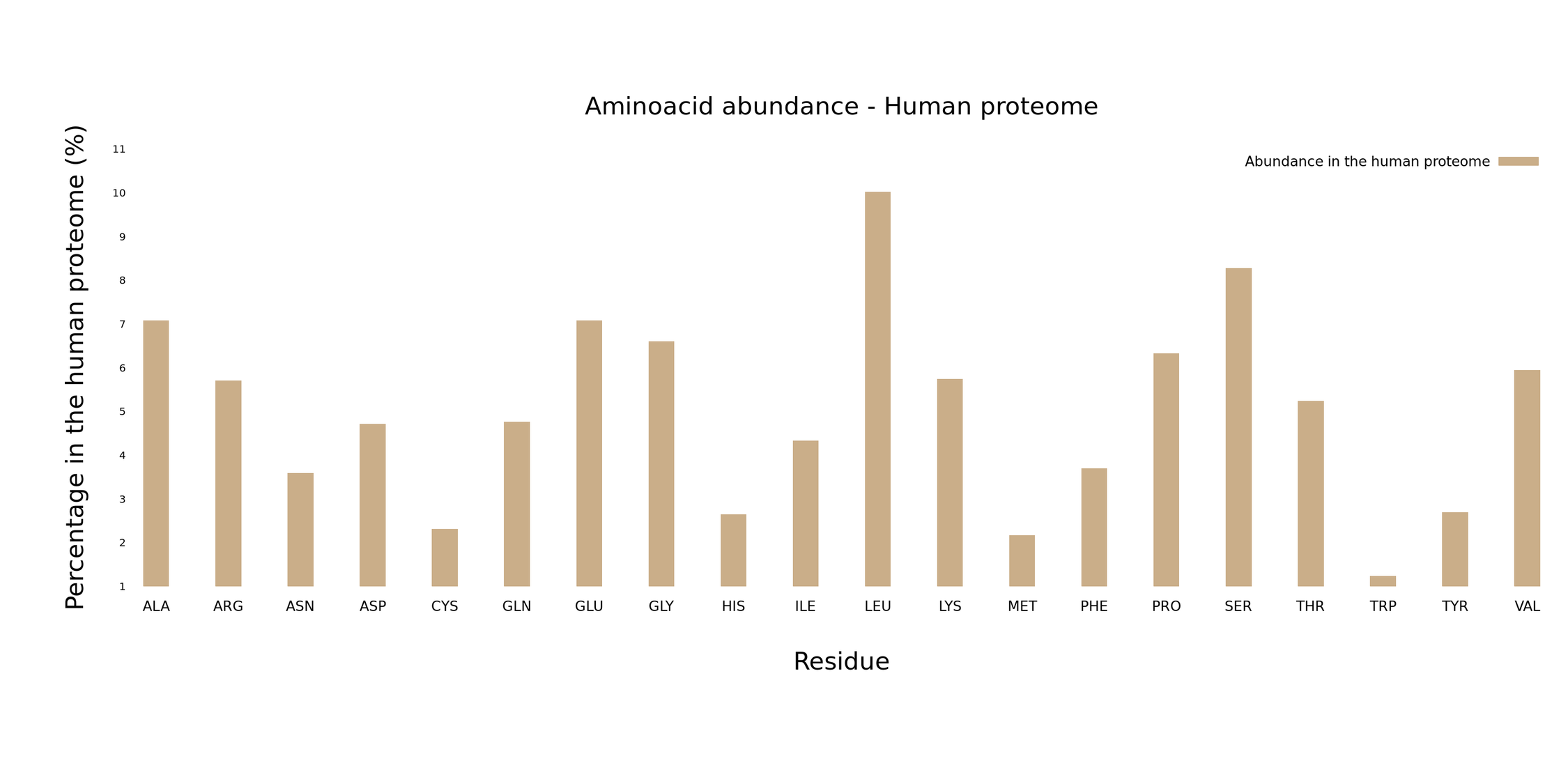

Supplement: S1 File — (ZIP) [file pone.0290890.s001.zip › Supplementary Information/FigS1.tif]
