# Supplementary material for: Expanding the repertoire of human tandem repeat RNA-binding proteins
Source: PLoS One. 2023 Sep 20;18(9):e0290890. doi: 10.1371/journal.pone.0290890 (PMC10511089; doi:10.1371/journal.pone.0290890)
Supplement: Attachment — Submitted filename: Response to the reviewers_AGB.docx [file pone.0290890.s002.docx]

**Resubmission of a revised version of PONE-D-22-25169**

Dear Dr Preiss,

We thank you very much for your last email, which contained the reviewer's opinions about the original version of our manuscript (which was submitted with manuscript number PONE-D-22-25169). I would like to apologise for the delay in getting the revision back to the journal. We are submitting as a new manuscript as advised by the editorial office. We have carefully read the reviewer and editorial comments and considered the suggestions and concerns. We thank both for the thoughtful reading and their observations and advice. As you will see in the response to reviewers, we have addressed their suggestions and responded to each point raised. The original reviewer comments are shown in italics and our responses are shown in dark blue text without italics. We believe the changes we introduced have improved the quality of the article, and its accessibility to a broader audience. We hope our manuscript is now ready for publication.

Yours sincerely

Alex Bateman

**Reviewer #1:**

*The research article entitled "Expanding the repertoire of human tandem repeat RNA-binding proteins" by Ormazabal et al. and Bateman describes a bioinformatic analysis intended to identify proteins within the human genome that might harbor novel tandem repeat RNA binding domains (trRBDs). The rationale behind this study is to build a database of such potential proteins, beyond those that have already been identified and studied, for experimental validation and possible applications that might benefit from the development of new sequence- or structure-specific RNA protein binding interactions.*

*The steps in the study appear to be comprised of (1) screening the alphafold database of human protein structural models (and corresponding sequences) for tandem repeats using a variety of repeat prediction servers; (2) screening the output of that step against the RBDmap database of RNA-interacting regions in human proteins (which were determined experimentally); (3) identifying overlap regions between predicted tandem repeat regions and RNA binding regions to create a list of candidate tandem repeat RNA binding motifs ('trRBDpeps').*

*The authors then go on to examine differences in the identification of potential trRBDpeps (and in the potential characteristics of those proteins) based on whether the tandem repeats were identified based on only sequence analyses (i.e. clear sequence identity between tandem repeats that are not readily identified in the predicted 3D structural model of the protein), structural analyses (i.e. significant structural similarities that are not readily identified at the amino acid sequence level (which happens readily if the underlying repeat topology can accommodate extensive sequence divergence) or both. They also devote text to modeling confidence for each protein, amino acid composition for tandem repeat regions that either are or are not implicated as being involved in RNA binding, and gene ontology functional annotations for the hits from their analysis.*

*In the end 219 human proteins are implicated as containing trRBDs (24 from sequence-based hits; 67 from structure-based hits, and 128 from both forms of identification analyses). The analysis does not apparently lead to an exhaustive list of such proteins, as at least one well-characterized trRBD (UniProtKB: P10155) is not identified.*

*Overall, this is a potentially interesting analysis that should be useful to investigators interested in either tandem repeat proteins and or RNA binding proteins. I have only minor queries, that are related to the presentation in the main text, which I find to be far too heavily oriented towards details of their bioinformatic analysis that (with all due respect) are not particularly interesting.*

*That text, which I strongly recommend condensing (see point #2 below) is presented at the expense of immediately and clearly telling an interested molecular biologist more about what's most interesting from a structure-function standpoint: What are the most 'novel', previously unidentified trRBDs and corresponding repeat motifs that laboratory investigators might wish to dive into? That information is made available in supplementary material, but a simple, top-level overview and anecdotal description of a two or three of the most interesting new 'hits' would be much, much more interesting and useful than all the space and text currently devoted (for example) to amino acid composition or pLDDT profiles.*

We thank the reviewer for these insightful considerations about our article and for considering it a potentially interesting contribution to the current knowledge in this field. We agree with their general observations about the paper orientation and agree with the suggestion to make a stronger emphasis on the results that of higher interest to biologists. In the following paragraphs, we will mention the changes we introduced to respond to those recommendations:

*1. Cases where repeats are identifiable via sequence analysis but not via examination of predicted folds via structure-based tandem repeat identification services are interesting. Either (1) the repeats are structurally diverged from one another to the point that they don't actually appear to be structural repeats (which would seem unlikely since they are identifiable based on sequence...sequences usually diverge more rapidly than do underlying structural topologies) or (2) the sequence-based identification(s) are giving false positives, or (3) alpha-fold is not as accurate as one would think, or (4) something else about the structural predictions is confounding the analysis. The statement "This would be expected if these repeats either had no regular structure or were organized in non-symmetrical ways that were not detected by structure based methods" is unsatisfying. Could the authors please illustrate such an example by showing me the sequence and the corresponding predicted structure of such a situation, so a reader can easily visualize this situation?*

These cases do tend to be examples of repeats found in disordered regions of proteins. Thus we included a representative example to illustrate this case. The manuscript now includes a final section with interesting examples. There we discuss the case of a false-negative prediction regarding structure-based methods corresponding to a protein (LRPPRC), which is predicted as repetitive via sequence-based predictors. Structural models show this protein possesses a clear structural pattern forming a large solenoid of alpha helices that was not detected by structural methods. We also noted that the structure-based algorithm REP2 is able to identify this structural tandem repeat if we relax the cutoff criteria adopted by the default settings. This observation suggests that the standard set of parameters employed with this method may be too strict in some contexts. We prefer this situation to the possibility of reporting proteins that are not true trRBPs.

*2. Overall, the paper provided considerable text about sequence-based parameters (LDDT scores, amino acid composition, gene ontology analyses) but doesn't provide 'up-front' examples and illustrations of truly novel trRBDs (which I would imagine investigators in this area would immediately be interested in examining and testing experimentally...any new RNA recognizing TR motif would be hugely interesting. I would recommend significantly cutting the verbiage currently in place about the identified trRBD sequence parameters, and instead get more to the meat of their analysis, which would be newly implicated RNA binding motifs that might be amenable to establishment of modular recognition codes.*

These suggestions, which broadly agree with those mentioned by Reviewer 2, are important because they will help to enhance the scope and readability of our work. As mentioned above we included a new section discussing one intriguing example for every subset explored in this work. Although the reviewer asked for these to be up-front, that did not flow well in the current manuscript, so they are placed after the computational analyses.

We include the description of three proteins from our dataset that were not previously reported as trRBP. However, it is worth noting that these are already reported as RNA-binding proteins from the original RBDpep manuscript. We highlighted that some of the repeats discussed do not constitute the typical instances of TR motifs, thus potentially representing novel tandem repeat RNA binding motifs. We also included new figures to clarify the structures adopted by these proteins, the position of their tandem repeats, and the regions predicted as repetitive by different methods. Since the pLDDT score discussion is necessary to understand the relationship between the degree of order and the positions adopted by the tandem repeats in every example, we decided to keep that section in the manuscript. The same holds for the amino acid enrichment to understand how some of the repeats discussed in the examples received particular attention. The second paragraph of the “Amino Acid Composition” section discusses a potentially novel bona fide repeat in our dataset. We hope that the inclusion of this section gives a new biological focus to the manuscript.

**Reviewer #2**

*This paper expands the identification of predicted tandem repeats in RNA binding proteins due to the increased availability of high quality protein structure prediction due to the introduction of AlphaFold. The authors use multiple algorithms to identify tandem repeats using either sequence based or structure based methods. They then analyze whether repeats are identified through sequence, sequence and structure or structure methods and characterize aspects such as amino acid composition and GO or Pfam terms in these groups. They note that there are limitations to these methods so not every instance will be found.*

*For a paper applying existing prediction algorithms, I think their data analysis is appropriate. The authors provide an extensive library of supplementary materials including tables of proteins, GO terms and Pfam designations. They also provide both a protein structure and a figure graphically showing output of multiple bioinformatic tools used to analyze the presence of repeats, overlap with RNA binding regions and degree of structuredness for every protein they examined. The journal explicitly requires all data to be provided - if this graphical representation is sufficient then they have done so; if the journal wants the output from each algorithm in a more raw format, then that would need to be added.*

*Overall the paper represents an extensive research effort that would be useful to the community, but I suggest several minor revisions.*

*1. You mention that this is timely because more new high quality structure prediction data is available, although not for every protein. How much has your new analysis added to previous work?*

Our analysis is the first we are aware of that provides a comprehensive analysis of tandem RNA-binding repeats. But all of the proteins were previously known as RNA-binding. Without checking all of the relevant literature which is very large it is difficult to tell precisely which proteins are already known as tandem repeat RNA-binding proteins. But there are certainly many examples in our work that we now discuss.

*2. Although the writing is clear and easy to understand, often rather than identifying a prediction based on what is already known that is driving analysis, the authors indicate that an analysis might be interesting. I think the paper could be improved by instead making predictions or hypotheses and then analyzing whether data support them or are surprising.*

*a. The article states: "We were interested in identifying if there exist differences among the characteristics of the proteins identified depending whether they have structural or sequential repeats." I think that you could actually hypothesize that there would be certain differences based on what is known about RNA binding and repeat sequences. I think it would be a good idea to instead propose what types of differences you would expect and whether or not they were confirmed by your analysis. For instance, knowing that some RNA binding proteins have disordered regions that become ordered upon protein binding, one would expect to find these tandem repeats only by sequence tools. Indeed, this is what they found.*

This is a good suggestion that will make the paper more engaging, We rewrote the sentence mentioned by the reviewer to present it as a hypothesis, also including the expected results.

*b. Similarly "We were interested in investigating whether the RBDpeps and tandem repeats possess different amino acid compositions compared to regions that are not repeated or involved in RNA binding." could be turned into a predictions of what amino acids you might expect to find in repeats or RNA binding regions. The analysis would then confirm or refute these predictions.*

As in the previous suggestion, we decided to rewrite the sentence mentioned by the reviewer to make explicit the hypothesis behind it and the results expected.
